# Seasonal and Meteorological Influences on Stroke Incidence and Outcomes in a Tropical Urban Setting: A 10‐Year Retrospective Study in Douala, Cameroon

**DOI:** 10.1029/2025GH001485

**Published:** 2025-12-29

**Authors:** Annick Melanie Magnerou, Daniel Massi Gams, Agnès Laurella Stevie Matega, Eric Lamou Bila Gueumekane, Victor Sini, Jacques Narcisse Doumbe, Callixte Kuate‐Tegueu, Yacouba Njankouo Mapoure

**Affiliations:** ^1^ Faculty of Medicine and Pharmaceutical Sciences University of Douala Douala Cameroon; ^2^ Neurology Department Laquintinie Hospital in Douala Douala Cameroon; ^3^ Department of Clinical Science Institut Supérieur de Technologie Médicale of Nkolondom Yaounde Cameroon; ^4^ Faculty of Medicine and Biomedical Sciences University of Yaoundé I Yaoundé Cameroon

**Keywords:** stroke, weather, influence, sub‐Saharan Africa

## Abstract

Climatic factors may influence stroke patterns, but data from sub‐Saharan Africa are scarce. This study assessed the relationship between weather variables and stroke incidence, severity, mortality, and hospital stay in Douala, Cameroon. A retrospective review was conducted using medical records from three referral hospitals in Douala from January 2011 to December 2020. Adults (≥18 years) with neuroimaging‐confirmed ischemic or hemorrhagic strokes were included. Weather data: temperature, humidity, wind speed, precipitation, atmospheric pressure, and sunshine duration, were obtained from the national meteorological agency (ASECNA). Associations between weather parameters and stroke‐related outcomes were analyzed using univariate and multivariate logistic regression. Among 1,349 stroke cases (mean age 61 ± 13 years; 53% male), 65% were ischemic strokes. Stroke incidence peaked during the long rainy season (*p* = 0.053). Severe strokes were associated with the long dry season (OR = 1.88), low precipitation (OR = 1.74), and low sunshine (OR = 0.62), while the long rainy season was inversely associated with severity (OR = 0.60). Mortality was higher during the short rainy season, linked to high temperatures (*p* = 0.046) and moderate rainfall (*p* = 0.04). Longer hospital stays were associated with the long rainy season (mean difference of 2.3 days, *p* = 0.01), and were influenced by high wind (*p* = 0.023), heavy rain (*p* = 0.013), and low sunshine (*p* = 0.002). Weather conditions significantly affect stroke incidence and outcomes. Climate‐informed public health strategies could improve stroke prevention and care in tropical regions.

## Plain Language Summary

Stroke is a serious medical condition that happens when blood flow to the brain is blocked or when a blood vessel in the brain bursts. Weather and seasonal changes can affect human health, but there is limited information on how these factors influence stroke in sub‐Saharan Africa. This study looked at how weather patterns like temperature, rainfall, wind, and sunshine are linked to stroke cases in Douala, Cameroon. Researchers reviewed hospital records of adult stroke patients over a 10‐year period and compared this information with local weather data. The number and severity of strokes varied with the seasons. More strokes occurred during the rainy season, while more severe cases happened in the dry season. Death rates were higher in the short rainy season, and longer hospital stays were linked to weather conditions like heavy rain and low sunlight. Weather and seasonal changes may influence not only when strokes happen, but also how severe they are and how patients recover. Understanding this connection can help improve stroke prevention and care, especially in tropical areas where the climate has clear wet and dry seasons.

## Key Points

- Seasonal variation significantly influences stroke incidence in Douala, with peaks during the rainy season
- High ambient temperature and humidity were associated with increased stroke admissions and in‐hospital mortality
- Understanding local climate‐stroke dynamics may help inform public health planning and optimize stroke prevention strategies

## 1 Introduction

Stroke, as defined by the World Health Organization, is “a rapidly developing clinical syndrome characterized by focal (or global) disturbances in cerebral function, lasting more than 24 hr or leading to death, with no apparent cause other than a vascular origin” (Aho et al., 1980). While this definition remains widely used, the American Heart Association and American Stroke Association consider it outdated, as it does not fully reflect advancements in understanding stroke etiology, clinical recognition, and the use of neuroimaging (Sacco et al., 2013). Stroke remains the second leading cause of death and the primary cause of acquired disability globally. Additionally, it is the second leading cause of dementia, with multiple contributing risk factors, including meteorological conditions (Aho et al., 1980; Akinyemi et al., 2021).

Douala, located on the Wouri River estuary at 17 m above sea level, experiences a tropical climate. The city's year is divided into four seasons: two rainy seasons (the long rainy season from May to August and the short rainy season from September to October) and two dry seasons (the long dry season from November to February and the short dry season from March to April). The annual average temperature is 26.2°C, with total annual rainfall averaging 3,702 mm (ONACC, 2019).

While many studies have examined the association between weather conditions and stroke occurrence in regions like North America, Europe, and Asia, there is limited data from Africa, particularly Cameroon (Ansa et al., 2008; Kintoki Mbala et al., 2016). This study aims to explore the impact of weather parameters on the incidence and outcomes of stroke in Douala, Cameroon.

## 2 Methods

This retrospective study was conducted by reviewing patient records from three referral hospitals in Douala: Douala General Hospital, Douala Laquintinie Hospital, and Douala Gynecological‐Obstetric and Pediatric Hospital, all of which have neurology services. Patient records were examined from 1 January 2011, to 31 December 2020. We included patients aged 18 and older who were diagnosed with ischemic stroke (I‐stroke) or hemorrhagic stroke (H‐stroke) confirmed via neuroimaging (CT scan or MRI). Records with missing data or those concerning transient ischemic attacks, subarachnoid hemorrhage, or cerebral venous sinus thrombosis were excluded.

Weather variables were obtained as daily averages and matched to the specific date of each patient's stroke onset, as recorded in hospital archives.

Operational definitions:

‐ *Temperature Classification*: Maximum temperature was categorized as low (<28°C) or high (>33°C), while minimum temperature was categorized as low (<20°C) or high (>26°C).
‐ *Humidity*: Relative humidity was considered high if above 50% and low if below 35%.
‐ *Stroke Severity*: Severe stroke was defined by extensive lesions and clinical signs leading to coma or death.
‐ *Hospital Stay Duration*: A prolonged hospital stay was defined as one exceeding 7 days.

Ethical approval was obtained, and weather data, including temperature, humidity, wind speed, and precipitation, were sourced from the Agency for the Safety of Air Navigation in Africa and Madagascar (ASECNA). Patient files were retrieved from the archives of the neurology and internal medicine departments across the three hospitals.

Data were analyzed using SPSS version 23.0. Categorical variables were represented as frequency (*n*) and percentage (%). Continuous variables were presented as mean ± standard deviation. The Student's *t*‐test and Fisher's exact tests were used to compare categorical and continuous variables. Univariate and multivariate analysis was performed to determine the weather parameters associated with stroke occurrence. The significance level was set at *p* < 0.05. Given the number of weather variables tested, we recognize the increased risk of type I error. While a Bonferroni correction was not applied due to the exploratory nature of the study and the intercorrelation between meteorological variables, key findings were further assessed using multivariate models to reduce the likelihood of spurious associations.

## 3 Results

A total of 1,349 stroke records were included in the analysis. The mean age of the participants was 61 ± 13 years, with males comprising 53% of the sample. I‐stroke accounted for 65% of cases, while H‐stroke comprised 35%.

**Figure 1:**
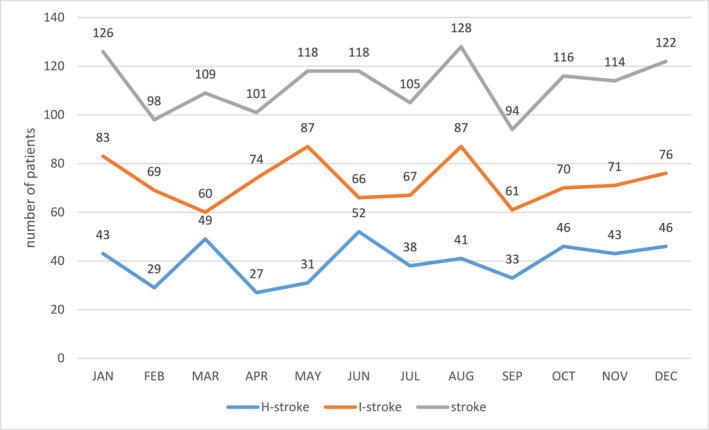
Monthly distribution of stroke cases by type occurrence (*p* = 0.053).

**Figure 2:**
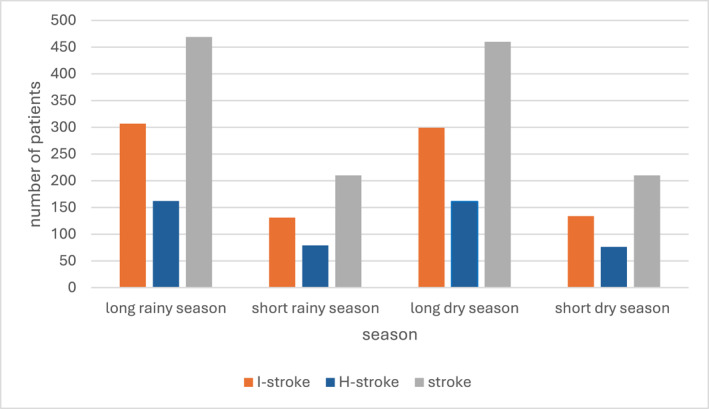
Seasonality of stroke occurrence.

*Seasonality*: The highest incidence of I‐stroke was observed in May (*n* = 87) and August (*n* = 87), coinciding with the long rainy season (Figure 1). H‐stroke peaked in March and June, during the short dry and long rainy seasons, respectively. Although August had the highest and September the lowest number of stroke cases, the overall seasonal variation did not reach statistical significance (*p* = 0.053) (Figure 2).

**Figure 3:**
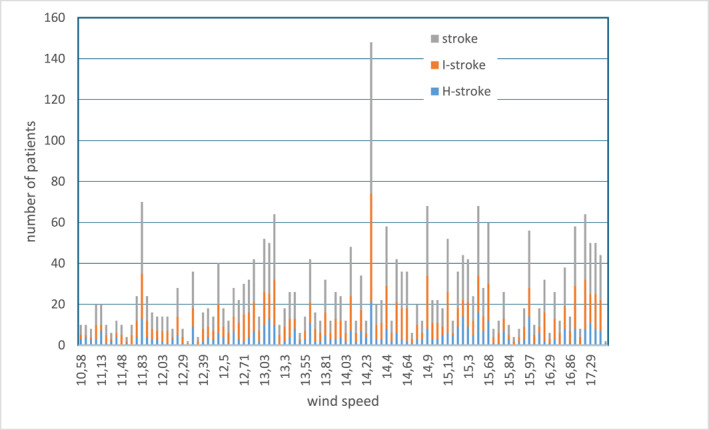
Distribution of stroke by wind speed.

*Wind Speed*: The median wind speed was 14.26 m/s, with I‐stroke more common during higher wind speeds and H‐stroke during periods of low wind speed (Figure 3).

**Figure 4:**
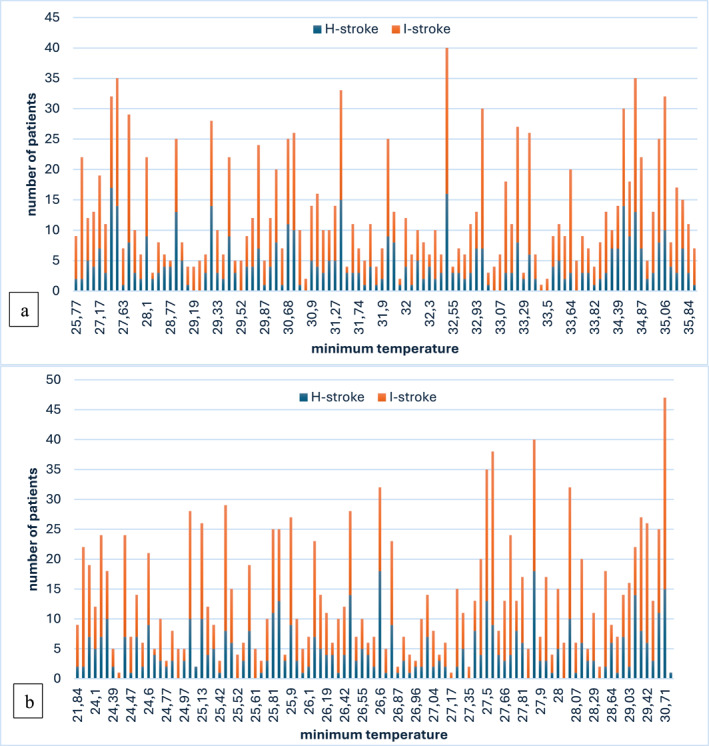
(a) Distribution of stroke by maximum temperature (*p* = 0.225). (b) Distribution of stroke by minimum temperature (*p* = 0.009).

*Temperature*: The frequency of I‐stroke was higher across all temperature ranges, but the maximum temperature did not show a significant association with stroke occurrence (*p* = 0.225). In contrast, higher minimum temperatures were significantly associated with I‐stroke (*p* = 0.009) (Figures 4a and 4b).

*Other Weather Parameters*: A significant association was found between stroke occurrence and variations in dew point (*p* = 0.035). Relative humidity levels also influenced stroke type, with low humidity linked to a higher frequency of I‐stroke (*p* = 0.003). No significant relationship was observed between stroke incidence and precipitation (*p* = 0.079), sunshine duration (*p* = 0.104), with air pressure (*p* = 0.08).

*Univariate and Multivariate Analysis*: Factors such as a maximum temperature between 28 and 33°C were found to be protective against stroke occurrence (*p* = 0.031, OR = 0.77), Table 1. Severe stroke was associated with low wind speed (OR = 1.54), low precipitation (OR = 1.74), and low insolation (OR = 0.62). The long dry season (OR = 1.88) was also strongly associated with severe stroke. Furthermore, a long rainy season (OR = 0.60) was inversely associated with stroke severity (Table 2). Longer hospital stays were associated with the long rainy season with a mean difference of 2.3 days compared to other seasons (*p* = 0.01).

**Table 1:** Factors Associated With Stroke Occurrence in Univariate Analysis

Variable	Category	I‐stroke (*n*)	H‐stroke (*n*)	OR (95% CI)	*p* value
Maximum temperature	High	313	148	1.25 (0.98–1.58)	0.072
	Normal (Ref)	425	263	0.77 (0.62–0.97)	0.031*
	Low	133	67	1.11 (0.81–1.52)	0.575
Minimum temperature	High	221	105	1.21 (0.93–1.57)	0.184
	Normal	439	257	0.87 (0.70–1.09)	0.225
	Low (Ref)	211	116	Reference	–
Wind speed	High	216	115	1.04 (0.80–1.35)	0.792
	Normal (Ref)	442	242	Reference	–
	Low	213	121	0.96 (0.74–1.24)	0.742
Humidity	Low	224	105	1.23 (0.94–1.60)	0.128
Precipitation	Low	361	185	1.12 (0.89–1.41)	0.354
Atmospheric pressure	Normal	471	234	1.23 (0.98–1.54)	0.077

*Note*. The bold values indicate statistical significance (p < 0.05).

**Table 2:** Factor Associated With Severity of Stroke

Variable	Category	Severe stroke (*n*)	Mild/moderate stroke (*n*)	OR (95% CI)	*p* value
Minimum temperature	Normal	98	598	1.75 (1.23–2.47)	0.002
	High	27	299	0.64 (0.41–0.99)	0.045
Wind speed	Low	50	284	1.54 (1.07–2.22)	0.022
Precipitation	Low	81	465	1.74 (1.24–2.44)	0.002
	High	43	450	0.64 (0.44–0.93)	0.021
Insolation	Low	26	295	0.62 (0.39–0.96)	0.034
Atmospheric pressure	Low	50	273	1.62 (1.13–2.34)	0.012
Season	Long dry	73	387	1.88 (1.34–2.64)	<0.001
	Long rainy	39	430	0.60 (0.41–0.88)	0.009

## 4 Discussion

This study is among the first to explore the relationship between weather variables and stroke epidemiology in an equatorial African setting, specifically Douala, Cameroon. The findings provide new insights into how climate parameters—including temperature, humidity, wind speed, and precipitation—may influence the occurrence, severity, mortality, and duration of hospitalization in stroke patients.

### 4.1 Stroke Type and Seasonal Variation

I‐stroke accounted for the majority (65%) of stroke cases, consistent with global epidemiological patterns and studies conducted in diverse settings such as Germany and England (Lorking et al., 2020; Palm et al., 2013). The seasonal trend showed peaks in stroke incidence during the long rainy season, particularly in May and August. This temporal clustering may be attributed to climatic stressors such as increased humidity and fluctuating temperatures, which potentially influence vascular reactivity, blood pressure regulation, and coagulation dynamics.

Notably, H‐strokes peaked during transitional seasons: March (short dry season) and June (onset of the long rainy season) suggesting that sudden shifts in weather, particularly barometric pressure and temperature, may serve as physiological stressors that increase cerebrovascular vulnerability. While our findings did not show statistically significant seasonality (*p* = 0.053), the pattern aligns with literature from temperate zones indicating seasonal fluctuations in stroke incidence, although the direction and strength of associations differ by region (Kloss et al., 2012; Palm et al., 2013; Takizawa et al., 2013). Similar findings have been reported in Nigeria and Congo, where higher stroke incidence and severity were observed during dry seasons, aligning with patterns seen in our Cameroonian cohort (Owolabi et al., 2018; Tshikwela et al., 2015). Studies in Southeast Asia have also linked humidity and temperature variations to increased stroke admissions, suggesting shared global trends in tropical climates (Chen et al., 2023).

### 4.2 Meteorological Parameters and Stroke Occurrence

Our results show that certain weather parameters were significantly associated with stroke subtypes. High minimum temperatures were associated with increased incidence of I‐stroke (*p* = 0.009), suggesting that nocturnal heat stress might influence blood viscosity, dehydration, and sympathetic nervous system activity, thereby predisposing individuals to thrombotic events. This finding supports prior studies linking elevated ambient temperature with hypercoagulability and increased incidence of ischemic events (Liss & Naumova, 2019).

Conversely, low wind speed was more frequently associated with H‐stroke. This could be interpreted through the lens of reduced evaporative cooling and the potential for indoor overheating, both of which may raise blood pressure levels, a known risk factor for intracerebral hemorrhage. Wind speed has received little attention in stroke literature, but some studies propose that its effect on human thermoregulation may indirectly impact cardiovascular stability (Cao et al., 2016).

Low relative humidity and dew point fluctuations were also significantly correlated with stroke incidence (*p* = 0.003 and *p* = 0.035, respectively). These variables can influence intravascular volume regulation and mucosal barrier integrity, particularly in the elderly and hypertensive populations, potentially increasing susceptibility to cerebrovascular events. High relative humidity may also exacerbate preexisting cardiovascular conditions by reducing the efficiency of heat dissipation, increasing thermoregulatory strain.

Interestingly, precipitation was not significantly associated with stroke occurrence (*p* = 0.079), although trends suggest a slight elevation in I‐stroke frequency during high‐rainfall periods. This may indicate that rainfall acts as a proxy for other variables such as barometric pressure, temperature variability, or reduced physical activity due to poor outdoor conditions.

### 4.3 Stroke Severity and Climatic Influences

We observed that severe strokes were more likely to occur during the dry seasons, particularly the long dry season (OR 1.88; *p* < 0.001). Dehydration, common during prolonged hot and dry periods, is known to elevate hematocrit and plasma viscosity, thereby increasing the risk and potential severity of thrombotic events (Liss & Naumova, 2019). Furthermore, high daytime temperatures, compounded by low humidity, may cause hemodynamic instability and vascular endothelial stress, compounding the risk of both ischemic and H‐strokes.

These results parallel findings from Liu et al. in China (Liu et al., 2018), who reported that cold seasons were associated with more severe strokes and worse short‐term outcomes. While the seasonal associations differ between temperate and tropical climates, the unifying hypothesis is that environmental stress (whether heat‐induced dehydration or cold‐induced vasoconstriction) can increase the severity of strokes.

### 4.4 Weather and Post‐Stroke Mortality

The association between mid‐range temperatures (28–33°C) and stroke mortality (OR 1.39; *p* = 0.046) highlights the complexity of thermal stress. While extremely high or low temperatures are recognized as cardiovascular stressors, even moderate ranges can impact vulnerable populations. Our findings that mortality was more pronounced during the rainy season, particularly the short rainy season, may be linked to compounded environmental and healthcare delivery factors, including access to emergency services, transportation delays, or increased prevalence of comorbid infections during periods of high humidity and poor ventilation.

Global evidence supports our observations. For instance, studies in China and Spain have demonstrated increased stroke mortality during both high and low ambient temperature extremes, with variations by age, sex, and socioeconomic status (Royé et al., 2019; Yang et al., 2018). The pathophysiological mechanisms underlying this U‐shaped association include increased blood pressure reactivity, oxidative stress, sympathetic overdrive, and endothelial dysfunction during thermal extremes (Kleeberg et al., 2025; Liss & Naumova, 2019). Similarly, barometric pressure changes and thermal stress may trigger cerebrovascular instability via oxidative stress and autonomic dysregulation (Hassan et al., 2022).

Sex‐specific differences in mortality responses to temperature also warrant consideration. Higher female mortality during hot periods, as observed in other cohorts, may stem from physiological differences in thermoregulation, body composition, and cardiovascular adaptation. These distinctions emphasize the need for gender‐sensitive public health interventions during extreme weather periods (Royé et al., 2019).

#### 4.4.1 Weather and Hospitalization Duration

The duration of hospital stay was significantly longer during the rainy season, particularly during periods of heavy rainfall, reduced sunlight, and high wind speeds. This association may be mediated by increased stroke severity, the prevalence of comorbid infections, or logistical challenges in patient care continuity during heavy rainfall. Additionally, psychological and environmental stressors during prolonged wet seasons may contribute to slower recovery trajectories and increased rehabilitation needs.

Comparable results were reported by Lorking et al. in the USA, who found a 7.3% increase in hospitalization duration during colder seasons (Lorking et al., 2020). These patterns may also reflect hospital system strain or variations in post‐acute care availability during different seasons. We acknowledge that meteorological influences on stroke risk may exhibit lag effects; however, in this initial analysis, we focused on same‐day associations. Future studies will incorporate lagged exposures (e.g., 1–3 days prior) to better capture delayed physiological responses to climatic stressors.

## 5 Limitations

This study has several limitations. Notably, certain confounders such as indoor environmental conditions, socioeconomic status, and comorbid infections, could not be controlled due to the retrospective design. We also note that air pollution, which may co‐vary with meteorological conditions and independently impact stroke risk, was not measured in this study. Future work incorporating satellite‐based or ground‐level air quality indices may help refine these associations. Additionally, meteorological data were collected at the city level and may not reflect microclimatic conditions at the patient level. Nevertheless, the multicenter nature of the study and the relatively large sample size enhance the generalizability of our findings.

## 6 Conclusion

Given the observed associations, incorporating meteorological surveillance into stroke risk models in tropical regions is recommended. Public health strategies such as weather‐adaptive education campaigns, hydration awareness, and seasonal monitoring of high‐risk populations can help mitigate stroke incidence and improve outcomes. Healthcare providers should remain vigilant to environmental risk factors, especially during extreme weather events.

## Conflict of Interest

The authors declare no conflicts of interest relevant to this study.

## Data Availability

This study used anonymized hospital records of stroke patients (including demographics, clinical characteristics, and outcomes) collected from major hospitals in Douala, Cameroon, between 2010 and 2019. The hospital data used in this study are not publicly available due to patient confidentiality and institutional data protection policies. These records include sensitive health information that cannot be sufficiently anonymized to comply with ethical and legal standards for public sharing. However, access to the dataset may be granted upon reasonable request, subject to approval by the ethics committee of the University of Douala and the hospitals' data governance boards. Interested researchers may contact the corresponding author, Dr. Annick Melanie Magnerou (melanieannick@yahoo.fr), to initiate the data access request process. This process includes submission of a research proposal, a signed confidentiality agreement, and evidence of institutional ethics approval. The authors provide an English README and data documentation to assist users. Daily meteorological data (temperature, humidity, rainfall, and atmospheric pressure) were obtained from the Cameroon National Meteorological Office (Météorologie Nationale du Cameroun, 2025). These data are licensed under CC‐BY 4.0. Historical weather data are archived at the Cameroon National Meteorological Portal. According to Prime Ministerial Decree No. 93/700/PM of 11 November 1993, access to these data requires a written request to the Department of National Meteorology using the appropriate form. Additional climate data for Cameroon are also available via the World Bank Climate Knowledge Portal. The statistical analyses and data visualizations were performed using SPSS (Statistical Package for the Social Sciences), version 23.0. Figures were produced in Microsoft Excel version 22503.1401.6.0. The full code used for data cleaning, statistical analyses, and visualization in this study is publicly archived on Zenodo and accessible at (Magnerou, 2025). The repository contains all R scripts necessary to reproduce the results presented in this article. This archived version is permanently preserved and compliant with FAIR data principles.
